# Understanding static, dynamic and cooperative porosity in molecular materials†
†Electronic supplementary information (ESI) available. See DOI: 10.1039/c6sc00713a


**DOI:** 10.1039/c6sc00713a

**Published:** 2016-04-13

**Authors:** Daniel Holden, Samantha Y. Chong, Linjiang Chen, Kim E. Jelfs, Tom Hasell, Andrew I. Cooper

**Affiliations:** a Department of Chemistry and Centre for Materials Discovery , University of Liverpool , Liverpool L69 7ZD , UK . Email: aicooper@liverpool.ac.uk; b Department of Chemistry , Imperial College London , South Kensington , London , SW7 2AZ , UK

## Abstract

The practical adsorption properties of molecular porous solids can be dominated by dynamic flexibility but these effects are still poorly understood. Here, we combine molecular simulations and experiments to rationalize the adsorption behavior of a flexible porous organic cage.

## Abstract 2

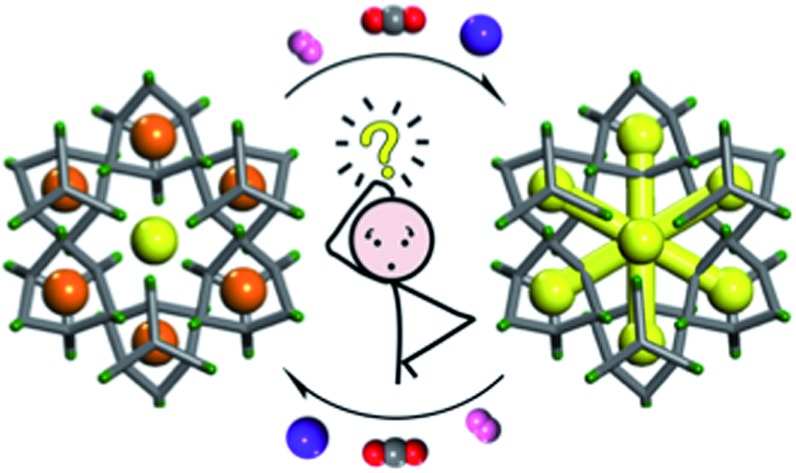
The practical adsorption properties of molecular porous solids can be dominated by dynamic flexibility but these effects are still poorly understood. Here, we combine molecular simulations and experiments to rationalize the adsorption behavior of a flexible porous organic cage.

## 

Most microporous materials (pores < 2 nm) are extended frameworks such as metal–organic frameworks (MOFs),^1^ covalent–organic frameworks (COFs),^2^ zeolites^3^ and microporous polymers.^4^ One can often understand their gas adsorption properties by computational analysis^5^–^7^ of the static framework structure: that is, the framework can be treated as rigid. A different, emerging class of porous materials is ‘porous molecules’, which includes calixarenes,^8^ cucurbiturils^9^ and porous organic cages.^10^,^11^ Here, consideration of flexibility is especially important because, unlike MOFs and COFs, the molecular building blocks in porous molecular crystals and glasses are not interconnected by strong covalent or coordination bonds. Indeed, flexibility is also important for extended frameworks, such as MOFs, where ‘breathing’^12^,^13^ or stimulus-response^14^ can lead to porosity that would be otherwise unexpected.^8^ Stimuli-responsive behaviour can arise from phase transitions of an entire lattice, which can be monitored crystallographically. Also, recent simulations of hydrocarbon diffusion in ZIF-8 demonstrated that framework flexibility can increase adsorbate self-diffusivities by several orders of magnitude in MOFs.^15^ Alternatively, diffusion in porous solids can be dominated by local, transient structural changes. These are not accompanied by a phase change, and they are therefore hard to study crystallographically. Such behaviour is also not accounted for by simulations based on the time- and volume-averaged crystal structure. As such, the diffusion of guests within porous molecular solids can be hard to understand and it is therefore challenging to design new materials for specific applications.

To understand diffusion behaviour in porous molecular crystals, it is vital that we consider the inherent structural motion of the porous host as well as the possible effects of cooperative host–guest interactions, as these have been shown to affect the practical sorption behaviour^16^ in applications such as the separations of organic molecules,^17^ noble gases,^18^ and chiral enantiomers.^18^

The definition of “porosity” for porous molecules is often nebulous, especially when the size of the guests approaches the size of the pores.^16^ In this study, we define the different kinds of porosity that exist for a flexible cage molecule, **CC2** (Fig. 1), which in turn provides a framework for classifying porosity in other porous molecular materials.

**Fig. 1:**
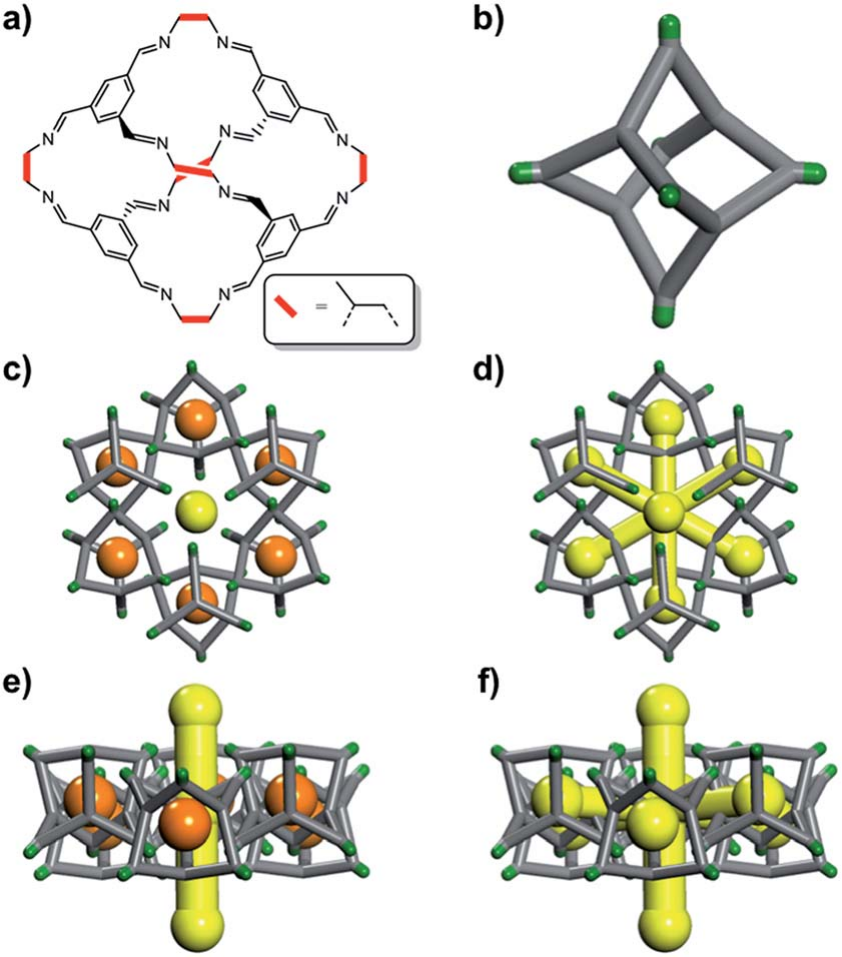
(a) Chemical structure of the porous cage, **CC2**; (b) simplified 3D representation of the **CC2** cage where vertices are coloured green. **CC2** forms window-to-arene stacks and the methyl vertices frustrate packing to give 1D inter-stack pore channels (yellow); the crystal structure also displays voids (orange), which encircle these 1-D channels as shown both (c) down the channels and; (e) orthogonal to the channels. Static porosity analysis, treating the molecules as rigid and immobile, shows that the voids and the 1D channels are disconnected (c and e). By contrast, dynamic and cooperative porosity analyses suggest that the cage voids can be linked to the channels, (d) and (f), but that the degree of linking is guest dependent.

The pores within a molecular crystal can be classified as either intrinsic or extrinsic.^19^ Internal voids within a molecule, such as an organic cage, are intrinsic pores. Extrinsic pores between molecules are a consequence of inefficient packing of the molecular subunits.^20^,^21^ It is possible to have both intrinsic and extrinsic porosity within the same molecular crystal. Recently, we reported a method that identifies transient channel formation between intrinsic and extrinsic voids in a porous organic cage crystal using molecular dynamic (MD) simulations and void decomposition.^18^ However, as we will demonstrate here, this method can miss rare events and cooperative guest–host effects, where the diffusing guest influences the host structure, resulting in a channel opening event.

We must first define the general term ‘porosity’. Barbour suggested that porosity can be divided into three types: conventional porosity, virtual porosity, and ‘porosity without pores’.^22^ Barbour showed methane sorption in a calixarene thought to be non-porous, and this was facilitated by the flexibility of the *tert*-butyl functional groups.^23^ To explore the role of host flexibility in ‘porosity without pores’, we divide porosity into three classes: static porosity, dynamic porosity, and cooperative porosity. These three classes can be considered in terms of decreasing pore interconnectivity (*i.e.*, static > dynamic > cooperative). Static porosity exists as a connected pore topology in the ‘static’ material, such that a probe is able to pass through the pores without requiring any host motion, and without the probe distorting the host framework. Dynamic porosity refers to a connected void network in the empty (*i.e.*, guest-free) host that exists only when the molecular and lattice flexibility is considered – that is, a pore network that is the result of inherent host flexibility, but which does not require the influence of a guest. Cooperative porosity refers to cases where the influence of a guest on the host is required in order to facilitate guest transport – that is, a material where voids are disconnected in the empty host, even when host flexibility is considered. These different types of porosity must always be defined with respect to a given probe radius or specific guest molecule: for example, a material might have static porosity for a small guest but cooperative porosity for a larger guest.

We illustrate these concepts here for a porous organic cage molecule, **CC2** (Fig. 1), which can pack in at least two polymorphic forms. We focus on **α-CC2**,^10^ which contains intrinsic cage voids that are disconnected from the extrinsic pore channels according to a ‘static’ porosity analysis, even for the smallest diatomic gas, H_2_. We use a combination of grand-canonical Monte Carlo (GCMC) sorption simulations, classical MD simulations, and *in situ* powder X-ray diffraction (PXRD) studies to establish if this lack of void connectivity really is the case, since it was postulated before that the isolated voids in **CC2** could in fact adsorb small gases,^10^ despite being formally disconnected in a static porosity analysis. Three guest molecules were chosen (H_2_, CO_2_ and Xe), which vary in shape, size, and charge distribution. H_2_ did not have sufficient electron density to allow structure solution, but *in situ* PXRD studies were feasible for the other, heavier gases, allowing direct observation of the preferred binding sites of CO_2_ and Xe in **CC2**.

In **CC2**, each molecular cage in the crystal (Fig. 1b) can exist as one of four positional isomers because of the disorder of the methyl groups over the vertex *exo* sites of the cage. It was therefore necessary to capture this in the simulations. To do this, ten models of a 2 × 2 × 2 **CC2** supercell were generated. In four of these models, the position of the methyl groups on each cage was kept constant (*i.e.*, an artificially ordered system). In the other six models, the position of each methyl group was randomized. Further information on this is given in the ESI,† Section 2.

To determine whether access to the intrinsic cage voids was required to explain the experimental gas uptakes in **CC2**, two types of GCMC adsorption simulations were run using the RASPA code^24^ (full details in the ESI†). In the first simulation, access to all voids was permitted. In the second simulation, the intrinsic voids were artificially blocked to simulate a case where these voids were inaccessible to guests. Subsequently, both the ‘empty’ and ‘blocked’ systems underwent 20 ns MD simulations, with an equilibrium period of 250 ps at 1 atm and 298 K using CSFF, a custom force field that we have previously developed for porous imine cages, including **CC2**.^25^ A 0.5 fs time step was used, with sampling taken every 1 ps.

The experimental gas sorption isotherms for H_2_, CO_2_ and Xe are shown in Fig. 2, along with the GCMC predicted uptakes. The isotherms were recorded at low temperatures to give close to saturated loadings for each gas. For H_2_ at 40 K, it is clear that the GCMC calculations do not require the formally isolated cage cavities to reach the experimental gas uptake (7.0 mmol g^–1^, Fig. 2a). Indeed, simulations where the intrinsic voids are blocked (6.4 mmol g^–1^) account well for the experimental uptake, and the ‘fully open’ simulation (10.4 mmol g^–1^) strongly overestimates the uptake. Next, MD simulations of the empty host were used to further investigate the connectivity between the channels and voids. The connected void space within the ten models was highlighted using a 1.09 Å probe,^16^ and these models were then superimposed. Interestingly, 8 of the 10 models showed formation of transient channels between the extrinsic one-dimensional channel and some of the intrinsic cage voids (for information, see the ESI,† Section 3–7). This highlights the influence of flexibility in the molecular crystal. The two models exhibiting only isolated voids within the cage cavities, post-MD, share a common methyl group in a specific position on each of the cage subunits (Fig. 3a). Hence, it seems that this methyl group, if artificially ordered in the simulation, ‘blocks’ the transient channel between the intrinsic and extrinsic regions, thus restricting the porosity. Although partial occupancies for the methyl groups in the single crystal X-ray diffraction structure indicate that this position is favored, it is not exclusively occupied.

**Fig. 2:**
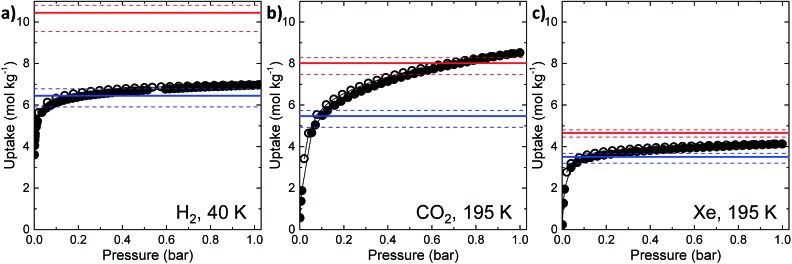
Simulated and experimental gas sorption results for **CC2** for (a) H_2_, (b) CO_2_, and (c) Xe. Experimental measurements are represented as black, filled circles (adsorption) and empty circles (desorption). Simulated uptakes at 1 bar are given as red lines (all sites accessible) and blue lines (intrinsic cage cavities blocked): the solid lines represent the average values of the 10 models, while the dashed lines indicate the maximum and minimum values in the set, respectively.

**Fig. 3:**
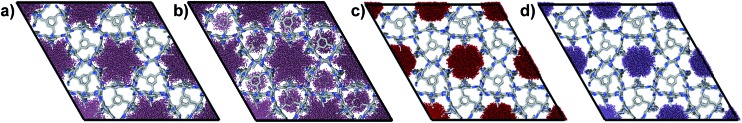
The empty host MD simulations were analysed using Zeo++ to highlight the accessible void volume determined for the guest-free **CC2** crystal structure. Superimposition of the H_2_-accessible void volume for (a) two artificially methyl-ordered models *versus*, (b) the methyl-disordered **CC2** models. Comparison of (a) and (b) shows how the relative positions of the methyl groups affect the guest accessibility of the cage cavities. The accessible void volume for (c) CO_2_, and (d) Xe for all 10 superimposed models; these simulations suggest that diffusion from channel to cage would not be observed for these large guests on the MD timescale, irrespective of the relative methyl group positions. The radii used were H_2_ = 1.09 Å, CO_2_ = 3.40 Å, Xe = 2.05 Å.

A disordered system was therefore used for the other 6 models (Fig. 3b). For these disordered models, diffusion of H_2_ from the extrinsic channel into the intrinsic voids was indicated by MD simulations (Fig. S13†). Hence, even at temperatures as low as 40 K, flexibility in the crystal can allow diffusion of H_2_ into the formally disconnected intrinsic cage voids, so long as there is not a methyl group located in a position that prevents this. We suggest that the experimental uptake deviates from the ‘empty’ simulation (Fig. 2a) because some fraction of the cage voids are inaccessible (*e.g.*, because a proportion of the methyl groups occupy blocking positions), and perhaps also because these diffusion events are so rare at 40 K that the isotherm shown in Fig. 2a is not fully at equilibrium. Overall, the available data would suggest that **CC2** exhibits at least some dynamic porosity with respect to H_2_ that allows the gas to diffuse into some proportion of the formally isolated cage voids.

For CO_2_, the experimental gas uptake in **CC2** was 8.1 mmol g^–1^ at 1 bar (Fig. 2b). Compared to the average values of blocked (5.4 mmol g^–l^) and the empty (8.0 mmol g^–1^) GCMC adsorption simulations, it is clear the GCMC values are much more consistent with a case where the CO_2_ molecules also occupy the intrinsic cage voids (Fig. 2b). However, superimposition of the MD analyses of all 10 empty host models showed separate extrinsic and intrinsic pore regions, as observed in the static single crystal structure (Fig. 3c). Hence, dynamic porosity was not observed during the 20 ns MD simulation, and even when the structure was loaded with CO_2_, the gas did not diffuse from the extrinsic region into the intrinsic cage voids over 20 ns simulations. The higher electron density of CO_2_ makes *in situ* PXRD studies of the gas-loaded structure feasible, allowing direct experimental observation of the sites that are occupied by this guest. Determination of the structure using PXRD data yielded six refinable CO_2_ positions – two inside the cage cavity, and four in the channel (Fig. 4a). All sites are fully occupied, although there are large refined displacement parameters, particularly for the CO_2_ positions in the extrinsic, one-dimensional channels. This suggests that cooperative porosity is indeed taking place, even though this is not captured in our 20 ns MD simulations, and this explains the close agreement between the ‘empty’ GCMC simulations and the experimental gas uptake (Fig. 2b). Unlike H_2_, the large quadrupole charge on each CO_2_ molecule provides strong intermolecular interactions, facilitating multilayer adsorption in the extrinsic channel.

**Fig. 4:**
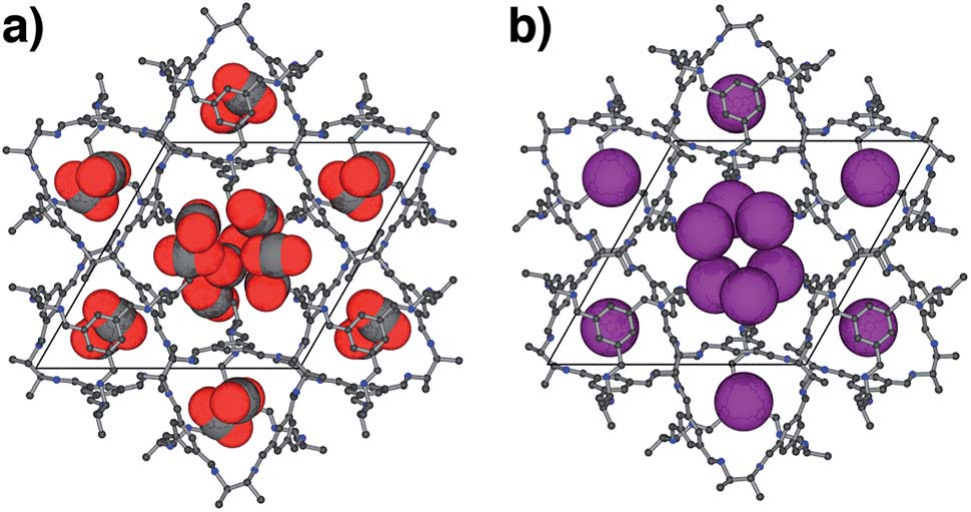
Crystal structures of (a) **CC2**·6(CO_2_) and (b) **CC2**·2.19Xe in **CC2** determined from *in situ* PXRD data. Cages are shown as ball and stick models (hydrogen atoms omitted) and guests as space-filling representations using van der Waals radii.

For Xe at 195 K and 1 bar, the experimental gas uptake in **CC2** is 4.1 mmol g^–1^, which lies between uptakes calculated from the ‘blocked’ and ‘empty’ GCMC simulations (3.5 and 4.66 mmol g^–1^, respectively). This could be interpreted as Xe diffusing into the extrinsic channels accompanied by partial but not full occupancy of the intrinsic cage voids. MD simulations of the empty host (20 ns) do not support this hypothesis, since no transient channels were formed that are large enough to accommodate Xe – again, the MD overlay shows clear separation between the two types of pores (Fig. 3d). In addition, no Xe was seen to diffuse from the extrinsic channel into the intrinsic voids throughout the 20 ns MD simulation when Xe was loaded into the extrinsic channel. However, as for CO_2_, the Xe-loaded structure determined from PXRD data shows unambiguously that Xe is present in both the extrinsic channel (53.6(4)% mean occupancy of three channel sites), and also in the intrinsic cage cavity (57.7(3)% of the cage sites occupied). This combination of simulation and PXRD again suggests that **CC2** exhibits cooperative porosity with respect to Xe.

## Conclusions

In summary, this study shows that a combination of techniques is needed to fully understand the nature of the porosity in these molecular materials. For **CC2**, specifically, the 1-D pore channel can be said to exhibit static porosity to all three gases studied, while the cage cavities show dynamic porosity to H_2_ but cooperative porosity to the larger gases, Xe and CO_2_.

This study also illustrates that MD simulations can strongly underestimate the porosity in porous molecular solids, since they can fail to capture rare diffusion events, and this is important for the design of new functional porous materials in the future. Enhanced sampling methods, such as metadynamics or umbrella sampling, may unlock new understanding of ‘porosity without pores’ in such materials. For example, we have showed recently that metadynamics can allow us to observe the rare cooperative diffusion event for SF_6_ in a porous organic cage host,^26^ while Sholl and co-workers have used umbrella sampling on a similar cage system, and discuss cooperative porosity of SF_6_.^27^ One limitation of these methods is that the diffusion pathway has to be pre-defined, this makes it which is hard to apply without prior knowledge of the system. Improved understanding of dynamic and cooperative diffusion events is essential if we are to design new porous molecular solids for difficult practical separations, in particular for guests such as Kr and Xe that differ only very slightly in size.

## Supplementary Material

Supplementary information
