# Supplementary material for: Understanding static, dynamic and cooperative porosity in molecular materials
Source: Chem Sci. 2016 Apr 13;7(8):4875–9. doi: 10.1039/c6sc00713a (PMC6016734; doi:10.1039/c6sc00713a)
Supplement: Supplementary file 1 [file SC-007-C6SC00713A-s001.pdf]

Electronic Supplementary Material (ESI) for Chemical Science.
This journal is © The Royal Society of Chemistry 2016

Supplementary Information

Understanding Static, Dynamic and Cooperative Porosity in Molecular Materials

Daniel Holden[†], Samantha Y. Chong[†], Linjiang Chen[†], Kim E. Jelfs,[‡] Tom Hasell[†], Andrew I. Cooper[†]

[†]Department of Chemistry and Centre for Materials Discovery, University of Liverpool, Liverpool L69 7ZD, U.K.

[‡]Department of Chemistry, Imperial College London, South Kensington, London, SW7 2AZ, U. K.

Contents:

1.0. **CC2** Isomerization
2.0. Static Porosity for **CC2**
3.0. Molecular Dynamic Setup
4.0. Dynamic Porosity
5.0. Grand Canonical Monte Carlo (GCMC) Simulation Setup
6.0. Gas filled Molecular Dynamics Simulations
7.0. **CC2** Synthesis and Adsorption
8.0. Powder X-Ray Diffraction
9.0. References

1.0. CC2 Isomerization

The **CC2** cage structure has fixed *exo/endo* sites, related to the staggered conformation of the propylene vertex groups. The *exo* site can be occupied, as the point away from the cage, reducing the steric interactions of the methyl group. There are six 1,2-propylene units within a single cage, and these exhibit positional isomerism – as such, the methyl group can occupy either of the two *exo*-positions, with hydrogens filling the remaining positions.

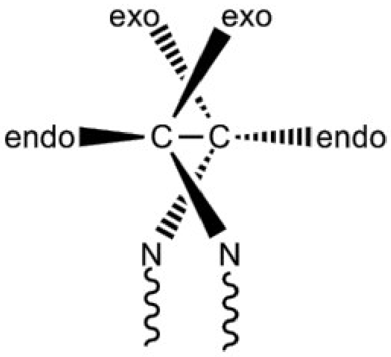

Figure S1. Diagram highlighting the different *exo/endo* positions available on each of the six **CC2** vertices.[5]

In total, the individual **CC2** subunit can exist in 4 isomeric forms, excluding enantiomers. Four of these have C_3 symmetry, and four have C_1 symmetry.

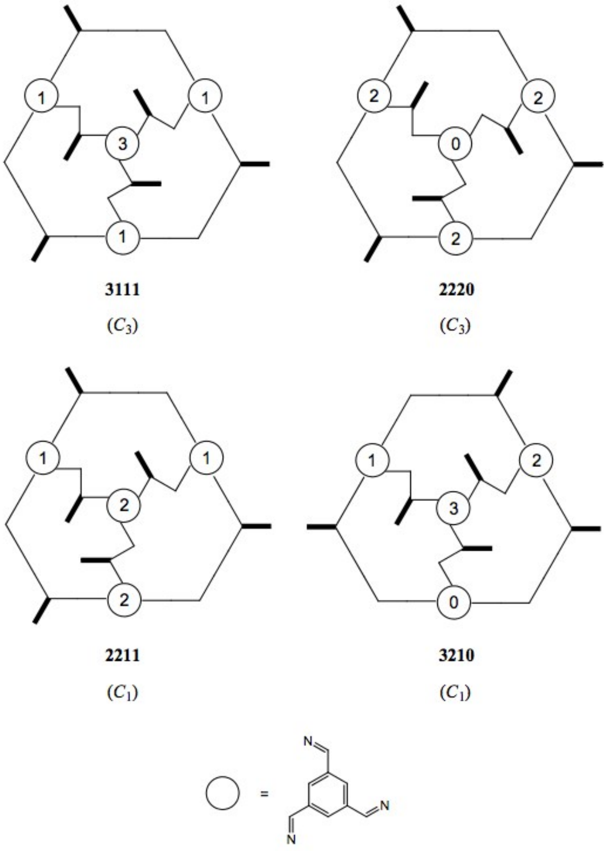

Figure S2. The four possible isomers of **CC2**, excluding enantiomers. The four-digit number reflects how many carbon atoms adjacent to each cage face $(C_6H_3(CN)_3$ units (3, 2, 1 or none; aryl faces shown as circles; methyl groups represented by bold lines).

To make a representative sample of these possible isomers, ten separate model systems were generated. This included four ordered systems, where each cage subunit was the same isomer, and also six models where the position of the methyl group was disordered. The placement of the methyl group in the disordered systems was randomized using the partial occupancies from the single crystal structure as a weighting factor.

Colour	Partial Occupancy
Red	0.765
Green	0.534
Blue	0.466
Orange	0.235

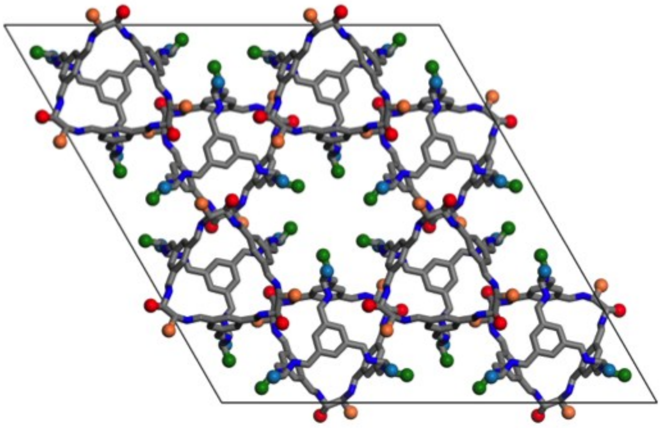

Figure S3. Partial occupancy of the methyl groups extracted from the X-ray single crystal structure, with their associated positions. Due to the imposed symmetry, the red-orange and green-blue *exo*-positions are mutually exclusive; *i.e.*, only one position can be chosen from each set.

For the four ordered systems, the sites the methyl groups occupy are as follows:

Ordered_01 = Red, Green
Ordered_02 = Red, Blue
Ordered_03 = Orange, Green
Ordered_04 = Orange, Blue

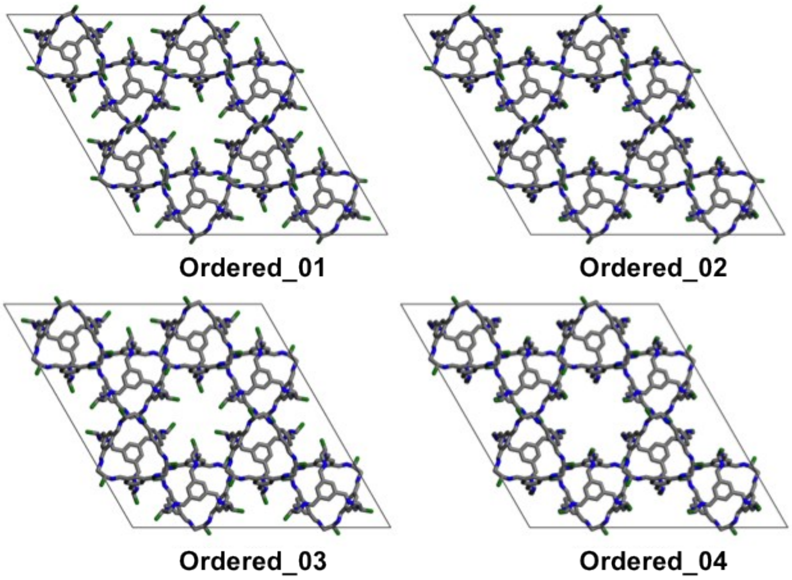

Figure S4. Four ordered systems of **CC2**.

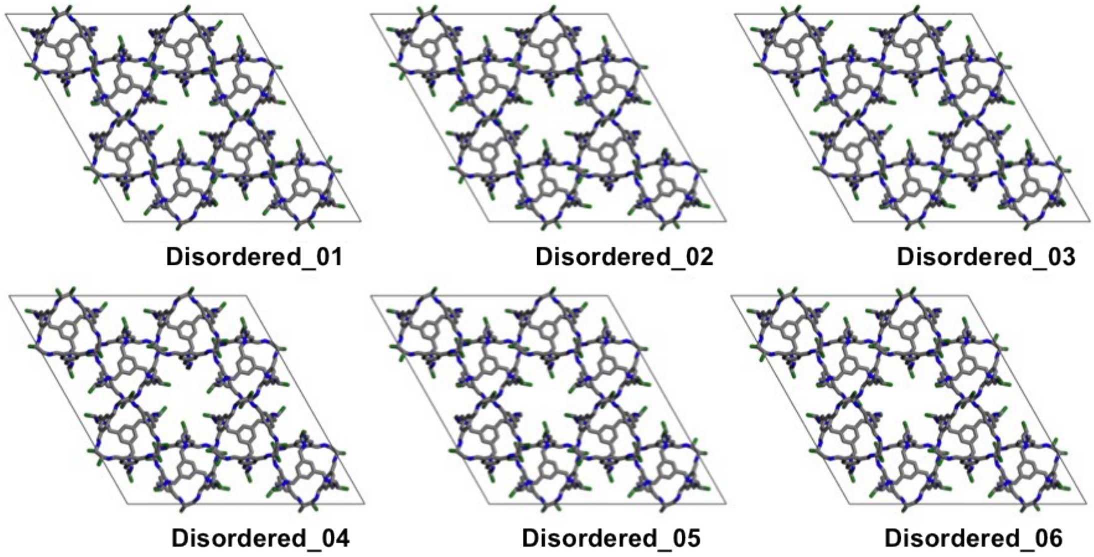

Figure S5. Six disordered systems of **CC2**, with the methyl group position randomized.

2.0. Static Porosity

The influence of the methyl group placement on the 'static' porosity for **CC2** is shown below. This highlights the importance of methyl group placement within the crystal, and suggests that one position (red, Figure S3) blocks a connecting channel between the one-dimensional channel and the cage cavity.

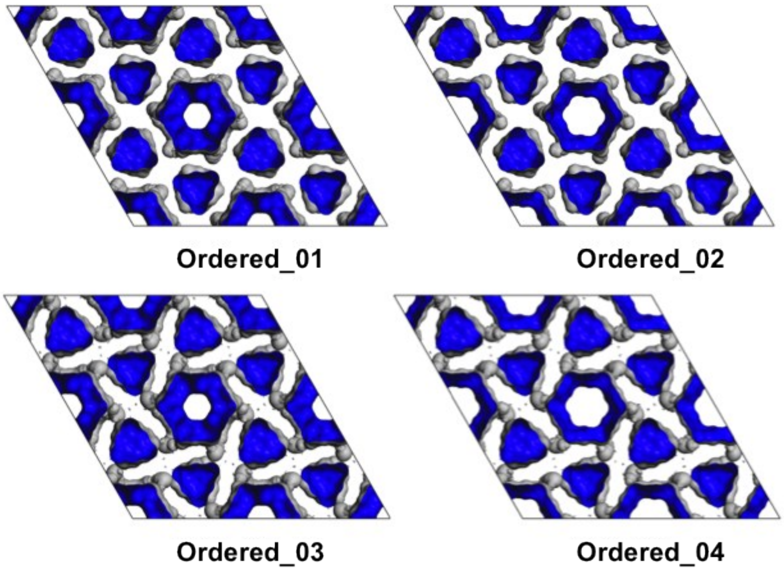

Figure S6. Four ordered systems of **CC2**, highlighting the static porosity using a hydrogen probe (1.09 Å).

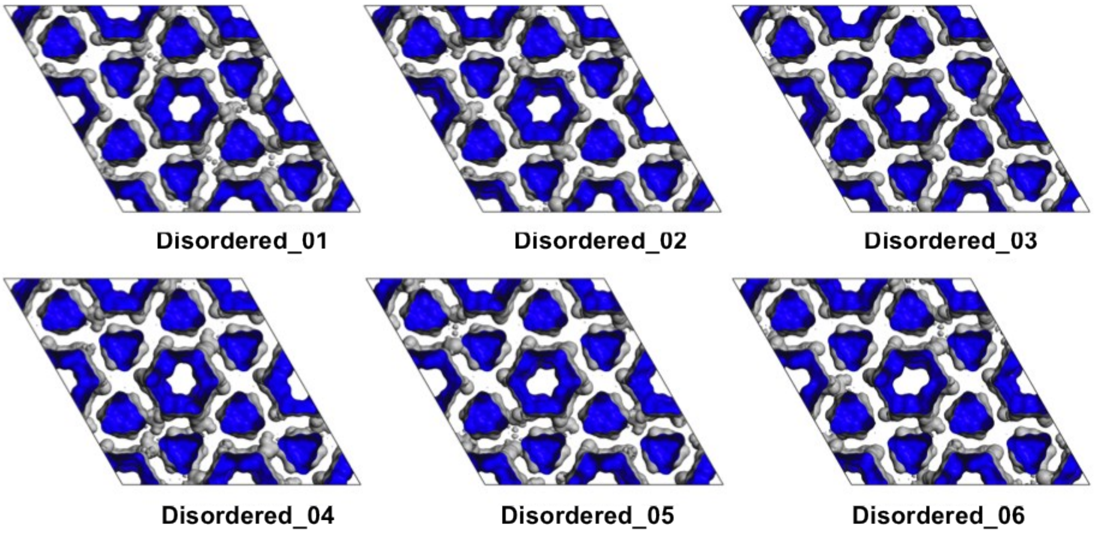

Figure S7. Six disordered systems of **CC2**, with the methyl group position randomized. The static porosity is displayed using a hydrogen probe (1.09 Å) is highlighted.

3.0. Empty Host Molecular Dynamics Setup

MD simulations of **CC2** were carried out using DL_POLY_2.20,[6,7] with a 2 × 2 × 2 supercell generated from the single-crystal structure data. Due to the disordered nature of the methyl group on the **CC2** vertices this was repeated for the ten models. A potential cut-off of 10 Å was used and electrostatic interactions were calculated using the partial charges from a cage specific forcefield (CSFF).[8] An NPT ensemble (constant number of moles, pressure and temperature) at 1 atm and 298 K was used with the Hoover barostat and thermostat,[9] and both had a time constant of 0.5 ps. A timestep of 0.5 fs was used, with the system first equilibrated for 50 ps with temperature scaling every 5 fs, followed by a production run of 20 ns, with a frame output every 1 ps. Four simulations were carried out on each model:

- MD of the empty system
- MD loaded with hydrogen in the 1-D channels
- MD loaded with carbon dioxide in the 1-D channels
- MD loaded with xenon in the 1-D channels

The empty systems were used to highlight the dynamic porosity of the system.

4.0. Dynamic Porosity

The accessible void volume plots for the 10 models were generated using Zeo++.[10] Probe radii corresponding to each gas (H_2 = 1.09 Å, CO_2 = 1.70 Å and Xe = 2.05 Å) were used to highlight the accessible pore regions over a sampling of 1,000,000 points. This was then visualised in the VisIt package,[11] and the 10 models superimposed, so that transient channel formation could be ascertained by inspection of the void histogram (Figure S8).

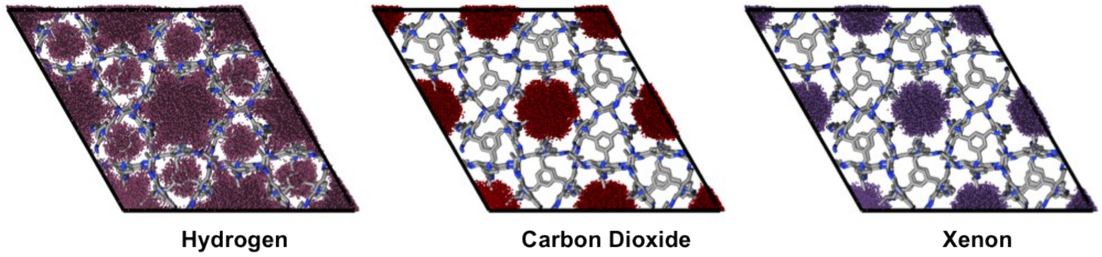

Figure S8. The accessible void volume for all 10 MD models superimposed to ascertain whether transient channels are accessible to a) H_2, b) CO_2, and c) Xe.

It was evident that transient channels between the internal cage cavities and the 1-D channel were only present for the H_2 simulations. Hence, the accessible void volume for each of the 10 models was plotted, Figure S9-10.

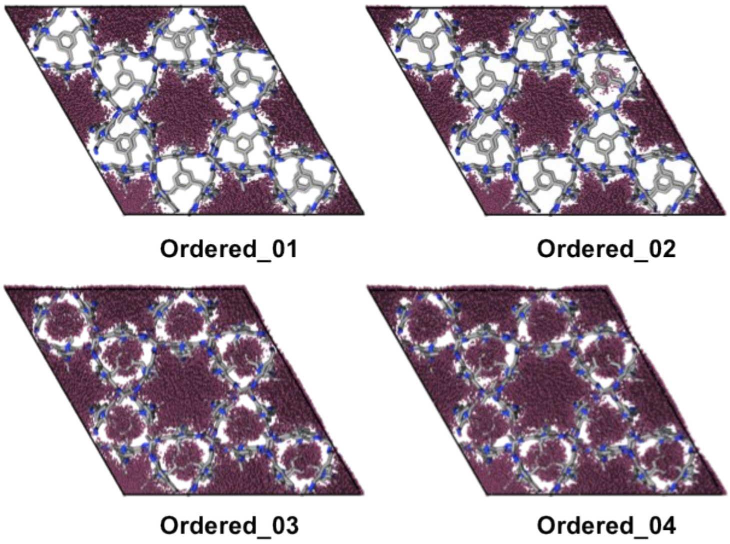

Figure S9. Four ordered systems of **CC2**, highlighting the dynamic porosity using a hydrogen probe (1.09 Å).

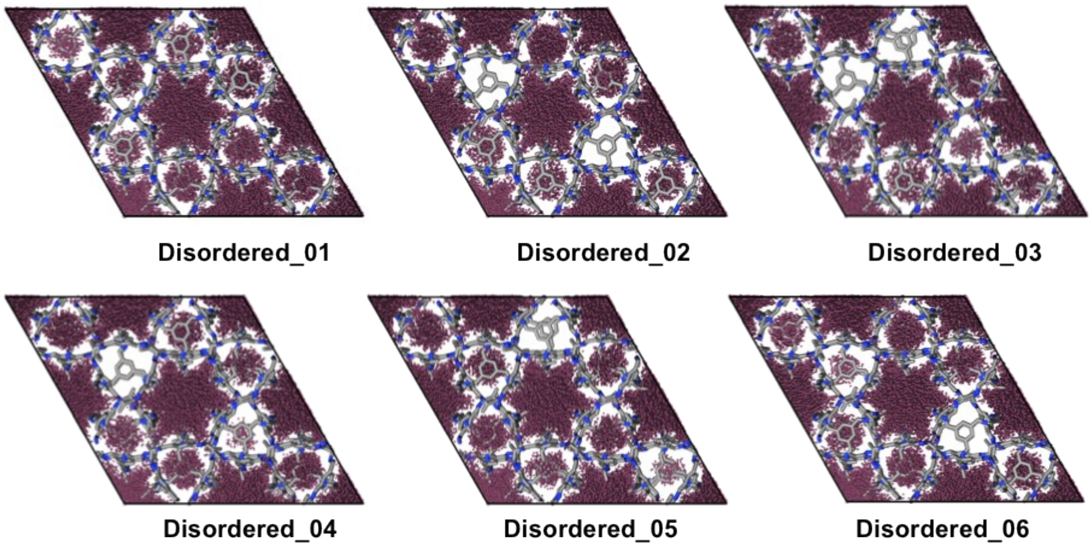

Figure S10. Six disordered systems of **CC2**, with the methyl group position randomized. The dynamic porosity is displayed using a hydrogen probe (1.09 Å) is highlighted.

5.0. Grand-Canonical Monte Carlo (GCMC) Simulations.

Gas adsorption in **CC2** was simulated using the GCMC method,[12] and the simulations were performed with the RASPA code.[13] In the GCMC method, the chemical potential, volume, and temperature are kept fixed while the number of gas molecules in the adsorbed phase is allowed to fluctuate so that the chemical potentials of the adsorbed phase and the bulk gas reservoir are equal. The input of a GCMC simulation includes the temperature and chemical potential of the gas molecules in the reservoir, and the output of the simulation is the average number of adsorbed molecules. This is analogous to an adsorption experiment, in which the temperature and bulk pressure of a gas are specified and the corresponding uptake is measured. The chemical potential, as used in GCMC simulations, can be related to the gas-phase pressure, as specified in experiments, by an equation of state. The Peng–Robinson equation was used here. The GCMC simulations performed in this work included a 100,000-cycle equilibration period and a 100,000-cycle production run. One cycle consisted of *n* Monte Carlo (MC) steps, with *n* being equal to the number of adsorbate molecules (*i.e.*, *n* fluctuated during the simulation). The trial MC moves – including insertion, deletion, translation, rotation, and reinsertion – were randomly selected with equal probabilities.

For GCMC simulations of CO_2 (at 195 K) and Xe (at 195 K), the host–guest and guest–guest (non-bonded) interaction energies were determined according to the Lennard–Jones (LJ) and Coulomb potentials, given as:

$$V_{ij} = 4\varepsilon_{ij}\left[\left(\frac{\sigma_{ij}}{r_{ij}}\right)^{12} - \left(\frac{\sigma_{ij}}{r_{ij}}\right)^{6}\right] + \frac{z_i z_j e^2}{4\pi\varepsilon_0 r_{ij}}$$

where ε_{ij} and σ_{ij} are the LJ potential-well depth and hard-sphere diameter, respectively; r_{ij} is the distance between atoms *i* and *j*; z_i and z_j are the fractional atomic charges; e is the elementary charge; and ε_0 is the vacuum permittivity. A cut-off radius of 12.0 Å was used for all LJ interactions (simple truncation), while all Coulomb interactions were computed using the Ewald summation technique with a relative precision of 10^{-6}. The Lorentz–Berthelot combining rules were used to calculate the LJ cross-parameters.

For simulations of H_2 adsorption at 40 K, quantum diffraction effects have to be taken into account, which was achieved here by using the so-called Feynman–Hibbs (FH) quantum effective potentials.[14] The quadratic FH potential, $U_{FH}(r)$, operates on a given classical model potential, $U_C(r)$, and is given by

$$U_{FH}(r_{ij}) = U_C(r_{ij}) + \frac{\hbar^2}{24\mu_{ij}k_B T}\left[U_C''(r_{ij}) + 2\frac{U_C'(r_{ij})}{r_{ij}}\right]$$

where $\hbar$ is the reduced Planck constant, k_B the Boltzmann constant, *T* the temperature, and μ_{ij} the reduced mass, which equates to $m_i m_i/(m_i + m_i)$ with m_i and m_j being the masses of the particles *i* and *j*. In our simulations, $U_C(r)$ took the form of LJ potential. Together with a simple charge-charge Coulomb potential, the total (non-bonded) interaction energy is given by

$$U^{tot}(r_{ij}) = 4\epsilon_{ij}\left[\left(\frac{\sigma_{ij}}{r_{ij}}\right)^{12} - \left(\frac{\sigma_{ij}}{r_{ij}}\right)^{6}\right] + \frac{4\epsilon_{ij}}{r_{ij}^2}\frac{\hbar^2}{24\mu_{ij}k_B T}\left[132\left(\frac{\sigma_{ij}}{r_{ij}}\right)^{12} - 30\left(\frac{\sigma_{ij}}{r_{ij}}\right)^{6}\right] + \frac{q_i q_j}{4\pi\varepsilon_0 r_{ij}}.$$

The forcefield parameters used for the adsorbates were taken from the literature and are summarized in Table S2, together with references to the sources. The OPLS-AA forcefield[15] was used to assign LJ parameters and partial atomic charges to the **CC2** atoms.

Table S2. Forcefield parameters for the adsorbates simulated and the **CC2** atoms.

adsorbate	ref.	atom type	ε/k_B (K)	σ (Å)	q (e)
**hydrogen**	16	$H(H_2)$	0.000	0.000	0.468
		$COM(H_2)$	36.700	2.958	-0.936
**carbon dioxide**	17	$O(CO_2)$	79.000	3.050	-0.350
		$C(CO_2)$	27.000	2.800	0.700
**xenon**	18	Xe	211.000	4.100	0.000

6.0. Gas filled Molecular Dynamics Simulations

The MD simulations, loaded with gas in the 1-D channels only (see GCMC simulation set up), were then analyzed, and it was evident that for CO_2 and Xe, the gases remained in the channel exclusively. However, for H_2, gas was seen to diffuse from the 1-D channel to the internal cage cavities via a transient channel. However, this transition was absent in two of the models; "Ordered_01" and "Ordered_02" (Figure S11d). These models share a common methyl position, and this position is blocking the path of the transient channel.

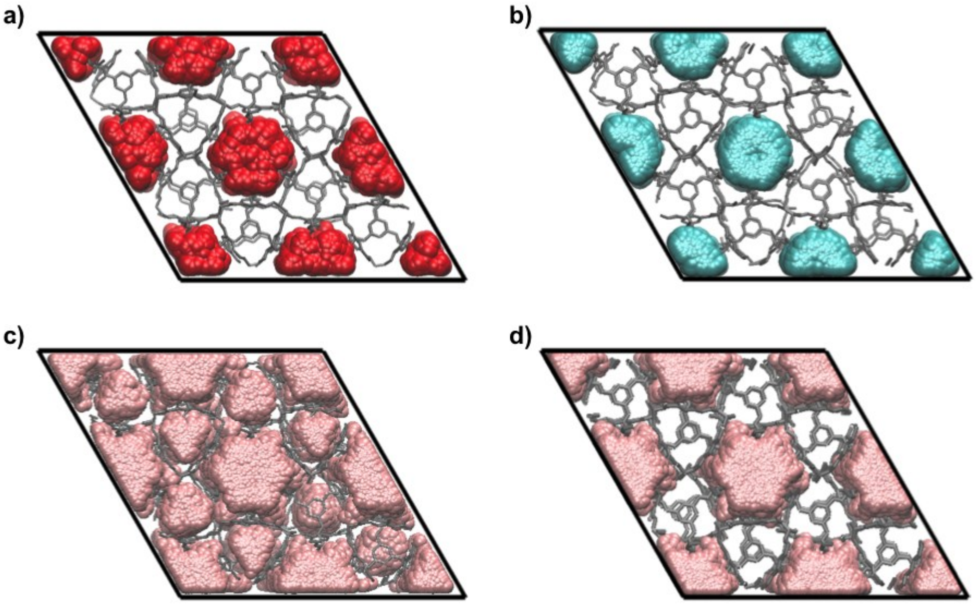

Figure S11. Superimposition of the MD trajectories for a) all 10 models of CO_2 in **CC2**; b) all 10 models of Xe in **CC2**; c) the 8 models for H_2 that show diffusion into the internal cage voids, and; d) the 2 models that only show diffusion down the 1-D channel.

7.0. CC2 Synthesis and Adsorption

CC2 synthesis: Six synthesis reactions were performed in parallel and under identical conditions. This was done to allow a comparison of reproducibility of gas sorption properties (Figure S12). For each sample: Acetonitrile (150 mL) was added slowly above 1, 3, 5-triformylbenzene (500 mg, 3.1 mmol) in a 250 mL round bottom flask at room temperature. After 5 minutes, a solution of 1,2-diaminopropane

(340 mg, 4.7 mmol) in acetonitrile (50 mL) was layered slowly into the round bottom flask. The resulting mixture was left covered for 60 h without stirring. A turbid solution was observed to form within 5 minutes after 1,2-diaminopropanewas added to the partially dissolved trialdehyde. This was followed by precipitation of a solid after around 5–6 h and, finally, pale white needles of **CC2** were observed to crystallize from solution after around 60 h. After 120 hours the crystals were filtered out to give the product at a mean yield of 57.1 % (standard deviation 4.7 %).

1H NMR (CDCl3, 400 MHz) δ 8.17-8.13 (m, 12H, -CH=N), 7.92-7.88 (m, 12H, -ArH), 4.09-4.06 (m, 6H, 6 x -NCH), 3.84 (m, 6H, -NCH), 3.52-3.47 (m, 6H, 6x -NCH), 2.01 (s, 3H nCH3CN),1.32 (d, *J* = 5.2 Hz, 18H, 18 x -CH3) ppm. 13C NMR (CDCl3, 100 MHz) δ 161.0, 160.9, 159.3, 159.1, 136.5, 129.7, 129.6, 129.5, 129.4, 104.6, 68.4, 66.8, 20.9, 1.9 ppm. IR (KBr pellet, v) 2967 (m), 2855 (s), 2360 (w), 1649 (s), 1446 (m), 1369 (m), 1252 (m), 1135 (m), 888 (w), 796 (w),692 (w) cm-1. ESI-MS (CH3OH) m/z: 877 [M+H]+ for C54H60N12, 899 [M+Na]+.

Gas Sorption Analysis: Surface areas were measured by nitrogen sorption at 77.3 K. Powder samples were degassed offline at 100 °C for 15 h under dynamic vacuum (10-5 bar) before analysis, followed by degassing on the analysis port under vacuum, also at 100 °C. Nitrogen and hydrogen isotherms, at 77 K, were measured using a Micromeritics 2420 volumetric adsorption analyser, allowing 6 samples to be measured in parallel to determine the variability. For measurements of most other gases, or at 273 K, a Micromeritics 2020 volumetric adsorption analyser was used, running one sample as a time with a water circulating chiller unit. Measurements at 195 K (CO_2) and 40 K (H_2) were performed using a Micromeritics 3flex surface characterisation analyser, equipped with a *Cold-Edge technologies* liquid helium cryostat chiller unit for temperature control.

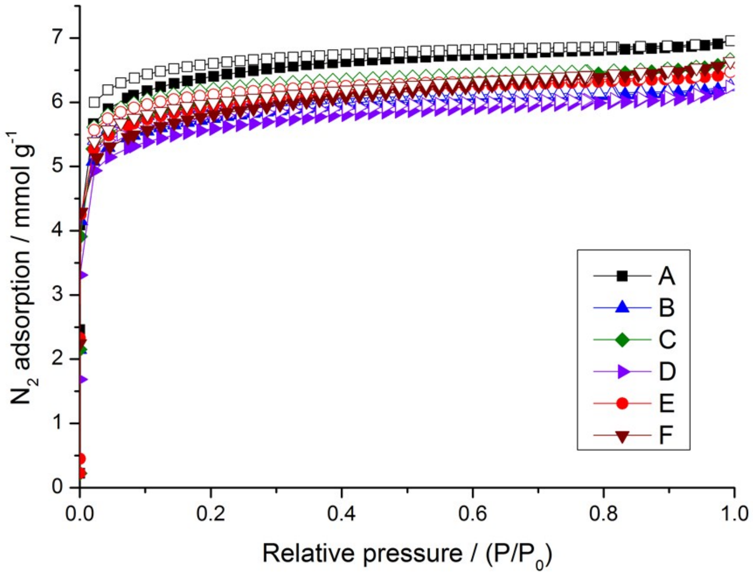

Figure S12. Nitrogen sorption isotherms, at 77 K and 1 bar, for 6 samples of **CC2**, run in parallel. Adsorption curves are shown as closed symbols and desorption as open symbols.

Table S1. Surface area and nitrogen uptake values and averages for the 6 parallel **CC2** samples.

CC2 sample	BET SA (m^2 g^{-1})	N_2 uptake (mmol g^1)
A	544.3	6.96
B	489.2	6.25
C	509.5	6.66
D	474.2	6.24

E	505.1	6.47
F	490.5	6.63
Mean and STD	502.13 ± 22.08 $m^2 g^{-1}$	6.54 ± 0.25 mmol g^{-1}

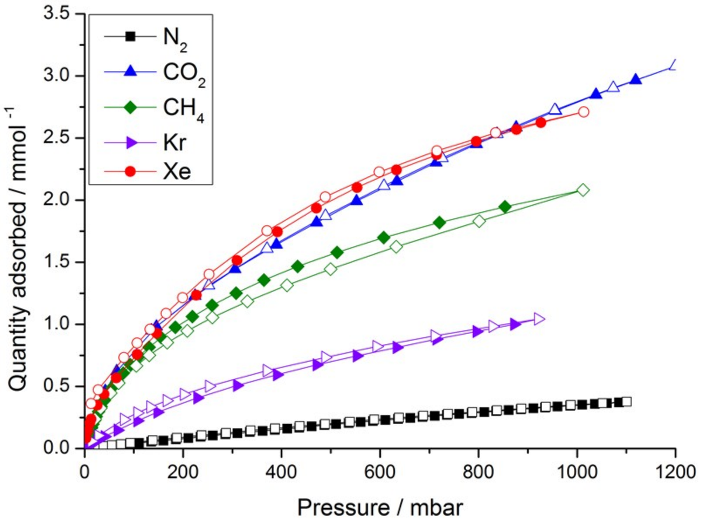

Figure S13. Gas sorption isotherms (273 K) for **CC2** with a variety of gases. Adsorption isotherms are shown as closed symbols and desorption isotherms as open symbols.

8.0. Powder X-Ray Diffraction

The gas adsorption of Xe and CO_2 in **CC2** was studied *in situ* using powder diffraction data collected at beamline I11 at the Diamond Light Source using the low pressure capillary gas cell.[19] Finely ground samples of **CC2** were dried in a vacuum oven at 80 ˚C for 12 hours, packed in 0.7 mm diameter borosilicate capillaries and mounted on the low-pressure capillary gas cell. Samples were activated by heating to 350 K using an Oxford Cryostream Plus under dynamic vacuum (approximately 10^{-5} bar). Data were collected using the Mythen-II position sensitive detector (PSD)[20] at 195 K for CO_2 and 298 K for Xe-loading experiments. An initial powder diffraction profile of guest-free **CC2** was collected for each sample under dynamic vacuum. The sample was rocked through ±15˚ in θ to improve powder averaging; nonetheless, a small number of sharp peaks in some datasets indicated the presence of large crystallites in the exposed sample. Where possible, a new section of the capillary was exposed. For each guest loading experiment, gas was dosed into the system in a number of pressure steps, up to a maximum of approximately 5 bar. Samples were allowed to equilibrate for a minimum of 30 minutes after gas was dosed into the cell. Several datasets were then collected using the PSD to confirm equilibration before increasing the pressure of gas in the system. The sample was then evacuated under dynamic vacuum to confirm removal of the guest from the pore structure.

Indexing, Le Bail and Rietveld refinement, and structure solution were carried out in *TOPAS Academic*.[21] *JANA2006*[22] was used to generate Fourier difference maps which were visualised in *VESTA 3*.[23]

The *in situ* PXRD data for CO_2 loading at 195 K exhibits small shifts of the diffraction peaks to lower angle with increasing gas pressure (Figure S14), corresponding to a small expansion of the unit cell to accommodate the CO_2 guest. The initial profile collected under vacuum showed extra diffraction peaks that could not be accounted for by the previously reported single crystal unit cell (Figure S15). The pattern was indexed and fitted with a triclinic *P*–1 cell (Figure S16) which corresponds to the loss

of threefold rotational symmetry in the parent *P*–3 structure. The triclinic symmetry persisted throughout subsequent guest loading. Lattice parameters extracted at each pressure step indicate that expansion of the cell to accommodate the guest occurs primarily between 0.5 and 1.1 bar (Figure S17). The main contribution to the modest increase in cell volume (3.1 % at 5.0 bar CO_2) is from expansion of the cell axes orthogonal to the extrinsic channel, i.e. parallel to the plane of the hexagonally-arrayed cage molecules. No significant change in the cell axis running parallel to the 1-D channel was observed.

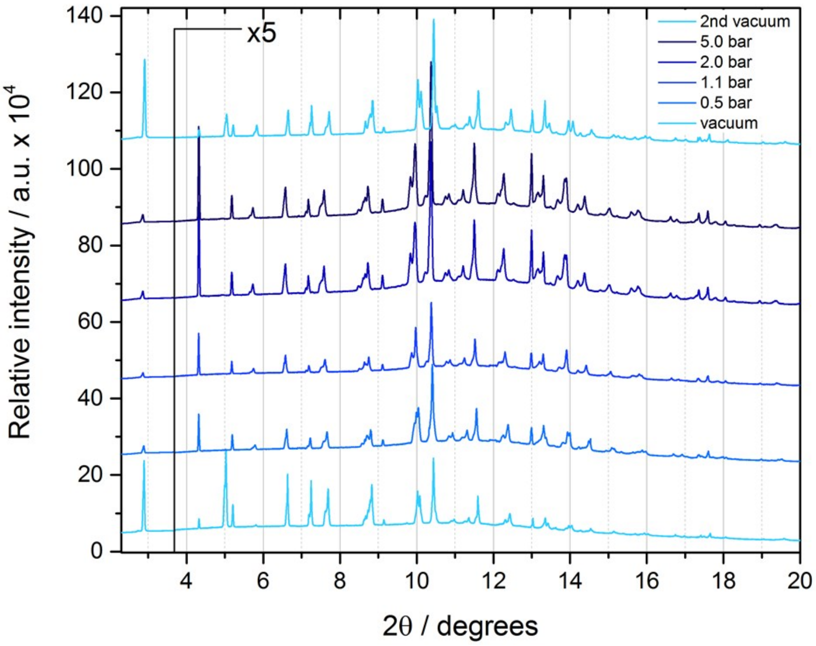

Figure S14. *In situ* powder diffraction data (λ = 0.825623 Å, 195 K) for **CC2** collected during CO_2-loading and subsequent removal under vacuum.

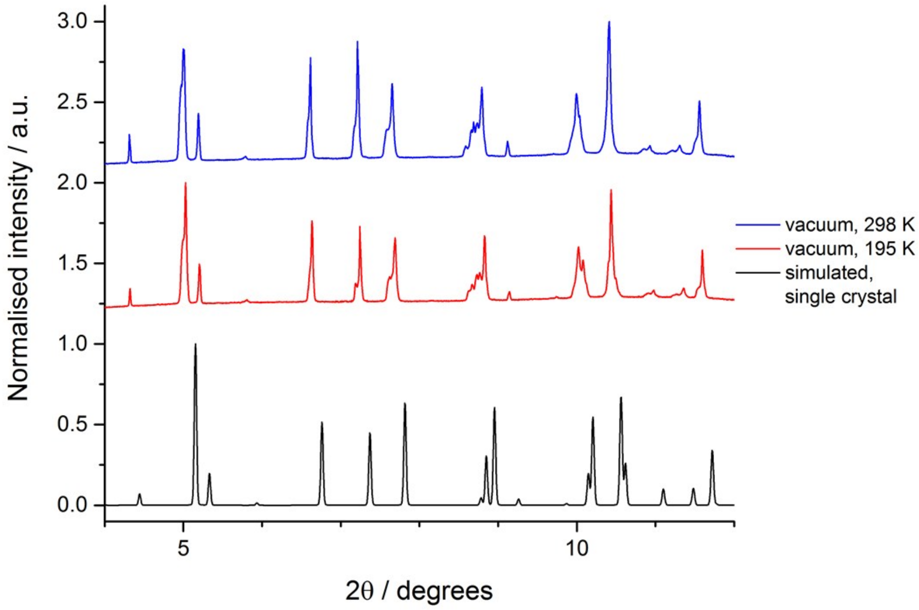

Figure S15. Comparison of the PXRD profiles collected on bulk powder samples of **CC2** under dynamic vacuum at 195 K (*red*) and 298 K (*blue*) with the pattern simulated from the single crystal structure (100 K, hydrated) (*black*). The experimental patterns show splitting of several diffraction peaks under vacuum, most obviously at $6 < 2\theta < 10°$, not accounted for by the unit cell and symmetry of the single crystal structure.

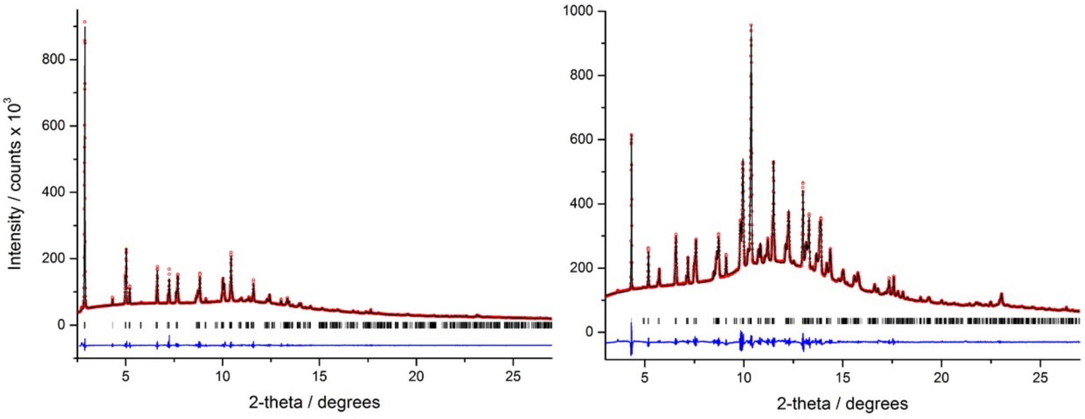

Figure S16. Observed (circles), calculated (solid line) and difference (below) X-ray powder diffraction profile for the Le Bail refinement of data for **CC2** collected under (*left*) dynamic vacuum (R_{wp} = 1.85 %, R_p = 1.10 %, χ^2 = 4.18) and (*right*) 5.0 bar of CO_2 at 195 K (R_{wp} = 2.09 %, R_p = 1.31 %, χ^2 = 8.00).

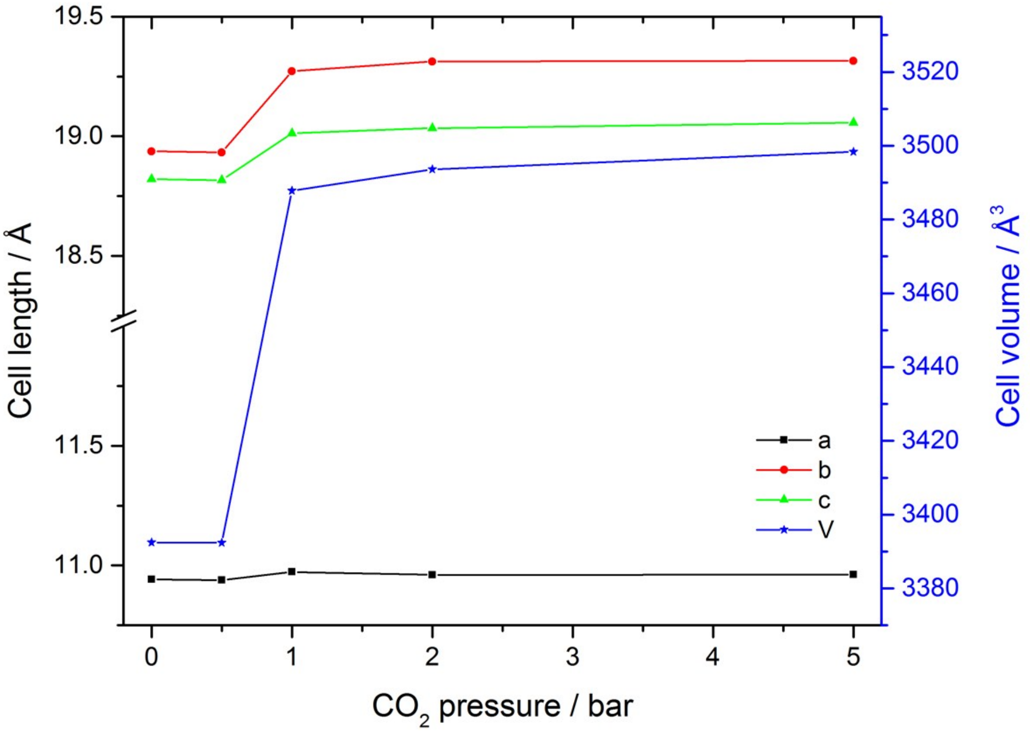

Figure S17. Variation of cell lengths and volume of CC2 exposed to increasing CO_2 pressure.

Direct space structure solution of **CC2** under 5.0 bar Xe was carried out using the simulated annealing algorithm implemented in *TOPAS Academic*.[9] The calculation included the full **CC2** molecule and a maximum of seven CO_2 molecules with variable occupancy. The calculation was run five times to

obtain a reproducible solution, which was used as the initial model for Rietveld refinement (R_{wp} = 2.29 %, R_p = 1.70 %, χ^2 = 8.84) (Figure S17) to obtain the final crystal structure of **CC2**·6(CO_2) (Figure S19). Geometric restraints were applied to all bond lengths and angles in the **CC2** molecule, derived from the published crystal structure of **CC2**.[3] It was not possible to refine the occupancies of methyl groups on the two *exo*-positions on each cage vertex. Therefore, they were modeled as fully disordered over the two possible positions on the vertices. During the refinement, some distortion of the vertices was encountered. This was overcome by strengthening restraints, but this may indicate that it was not possible to adequately model the positional disorder of the vertex groups from this dataset. Hydrogen positions were refined using the riding model. The CO_2 guests were treated as rigid bodies and exhibited large displacement parameters, suggesting their refined positions are likely to be more indicative of their general sites than exact location.

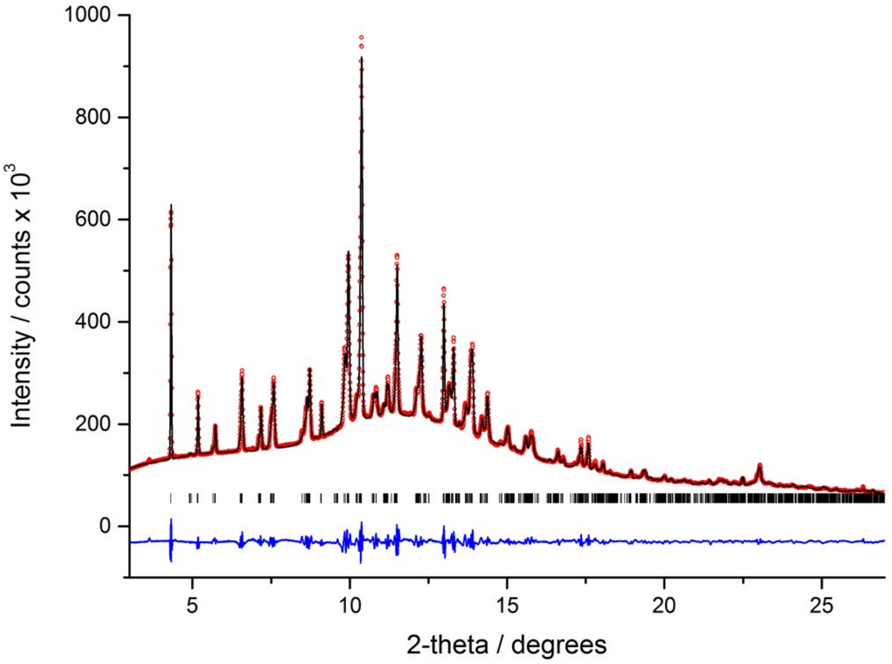

Figure S18. Observed (circles), calculated (solid line) and difference (below) X-ray powder diffraction profile for the Rietveld refinement of **CC2** under 5.0 bar of CO2 at 195 K (R_{wp} = 2.29 %, R_p = 1.70 %, R_{Bragg} = 2.19 %, χ^2 = 8.84). Reflection positions are also marked..

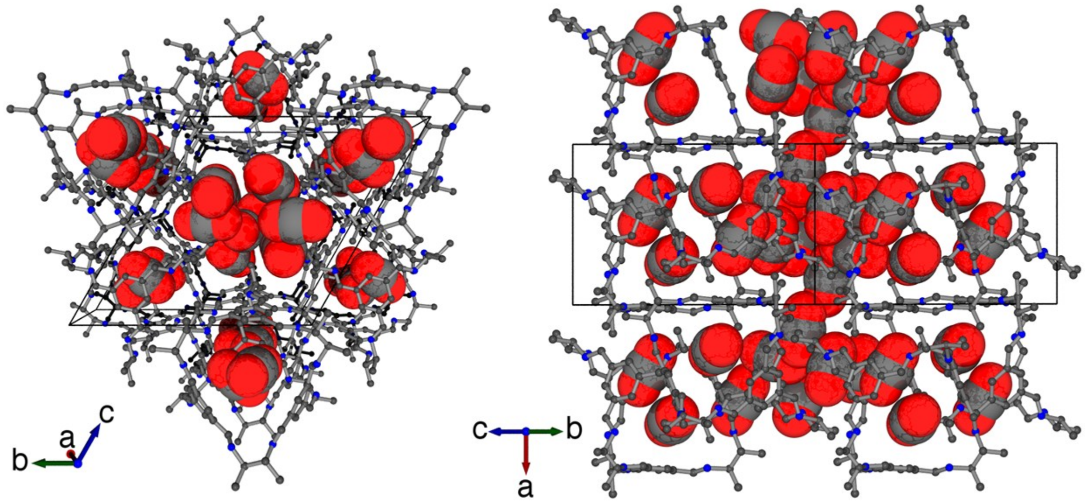

Figure S19. Refined crystal structure of CO_2-loaded CC2, **CC2**·6(CO_2) (a = 10.9651(1), b = 19.2912(7), c = 19.0581(4) Å, α = 119.861(3), β = 89.955(2), γ = 90.171(2)°, V = 3496.1(2) Å^3, P–1), drawn parallel to (*left*) the [100] direction and (*right*) the [011] direction. Cage molecules are drawn as ball-and-stick and carbon dioxide guest molecules in spacefilling representation. Hydrogen atoms are omitted for clarity.

Very small shifts in the peak positions are observed in the *in situ* PXRD data collected during Xe-loading up to 5.1 bar (Figure S20). Again, the single crystal lattice parameters could not account for the diffraction profile collected under dynamic vacuum. A cell corresponding to a small triclinic distortion of the original trigonal cell was found to index to the pattern. The lattice parameters extracted by Le Bail refinement (Figure S21) show that the axes orthogonal to the extrinsic 1-D channel contracted slightly with increasing pressures of Xe. The small contraction of the arrays of cages would result in slightly narrower channels, potentially improving interactions between cage hydrogens and Xe guests located in the channels. The overall magnitude of unit cell change was smaller than observed for the CO_2 guest (ΔV_{CO2} = 3.1 %, ΔV_{Xe} = 1.0 %). The axis running parallel to the extrinsic channel exhibits a very limited expansion (Δa = 0.2 % at 5.1 bar). The change with pressure also occurs more gradually than during CO_2-loading, with the most significant contraction occurring on loading up to 2.1 bar. The structure continues to adapt to guest exposure up to the maximum Xe pressure.

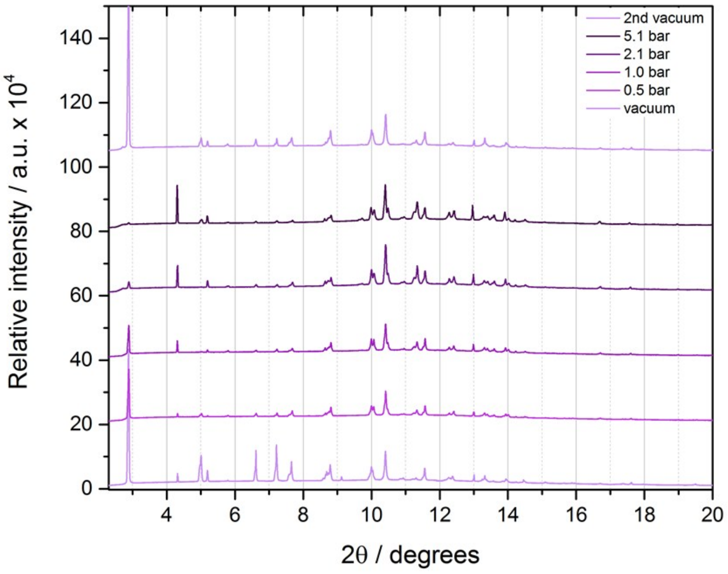

Figure S20. *In situ* powder diffraction data (λ = 0.825623 Å, 298 K) for **CC2** collected during Xe-loading and subsequent removal under vacuum.

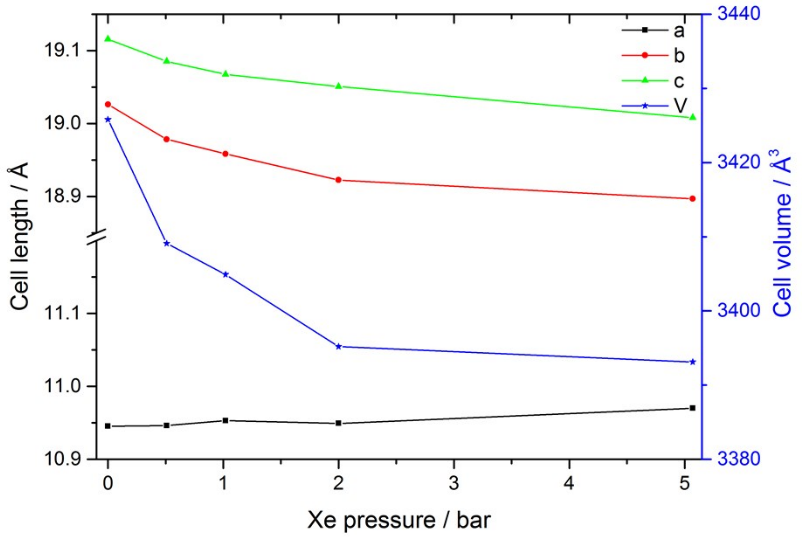

Figure S21. Variation of cell lengths and volume of **CC2** exposed to increasing xenon pressure.

The structure of evacuated triclinic **CC2** at 298 K was determined from the PXRD data collected under dynamic vacuum, using simulated annealing structure solution and subsequent Rietveld refinement. This model was then used to calculate Fourier difference maps based on the data collected at 5.1 bar xenon pressure (Figure S22). Electron density was observed in both the channel and the cage pore. This was confirmed by running simulated annealing calculations which included the full **CC2** molecule and five independent Xe atoms with variable occupancies. The calculations were run initially allowing both the cage and Xe atoms to translate freely throughout the unit cell. A set of five calculations was then performed with the cage positions fixed and the guest positions and occupancies allowed to vary freely. A xenon atom was located in the cage pore in all solutions and three or four Xe positions were identified in the channel. The best model with one xenon atom inside the cage and four in the channels was used as the initial model for Rietveld refinement (Figure S23), with the restraints as described for **CC2**·6(CO_2). As discussed previously, the methyl groups were modeled as fully disordered over the two possible positions on each vertex. Three xenon sites in the channel were retained in the final structure (Figure S24) with refined occupancies of 0.472(3), 0.564(3) and 0.572(4) and exhibited large displacement parameters. The xenon site in the care pore was 57.7(3) % occupied, resulting in total Xe refined occupancy of 2.19(1) xenon per **CC2** molecule.

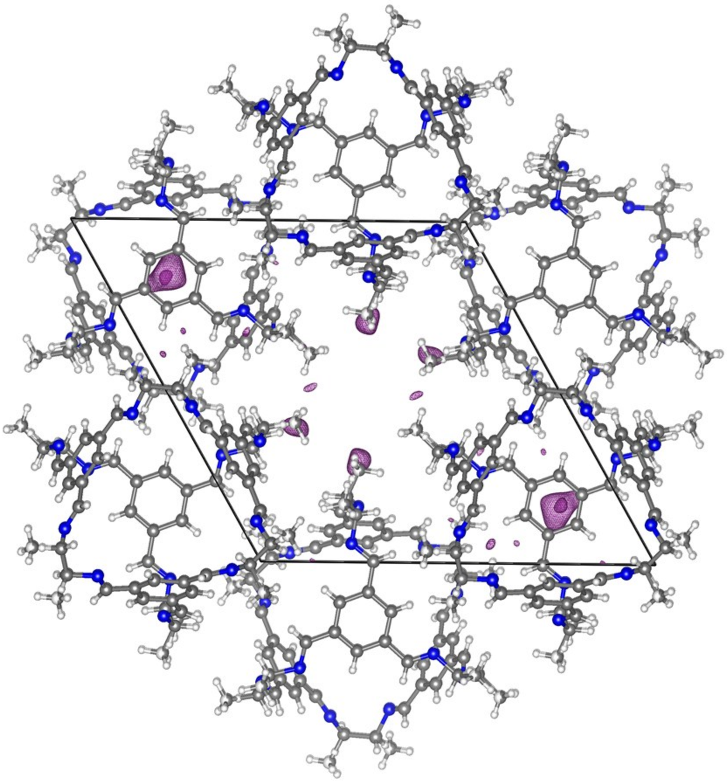

Figure S22. Three-dimensional difference Fourier map indicating regions of excess electron density in the **CC2** host structure loaded under 5.1 bar of xenon gas (isosurfaces drawn at 0.6 electrons Å^{-3}).

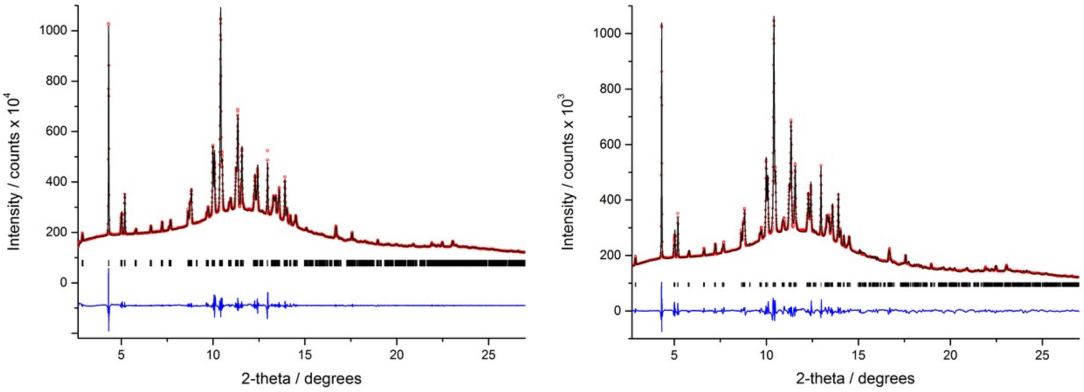

Figure S23. Observed (circles), calculated (solid line) and difference (below) X-ray powder diffraction profile for the (*left*) Le Bail (R_{wp} = 1.94 %, R_p = 1.05 %, χ^2 = 8.81.) and (*right*) Rietveld refinement (R_{wp} = 2.15 %, R_p = 1.50 %, R_{Bragg} = 2.56, χ^2 = 9.76) of PXRD data of **CC2** collected under 5.1 bar of Xe at 298 K.

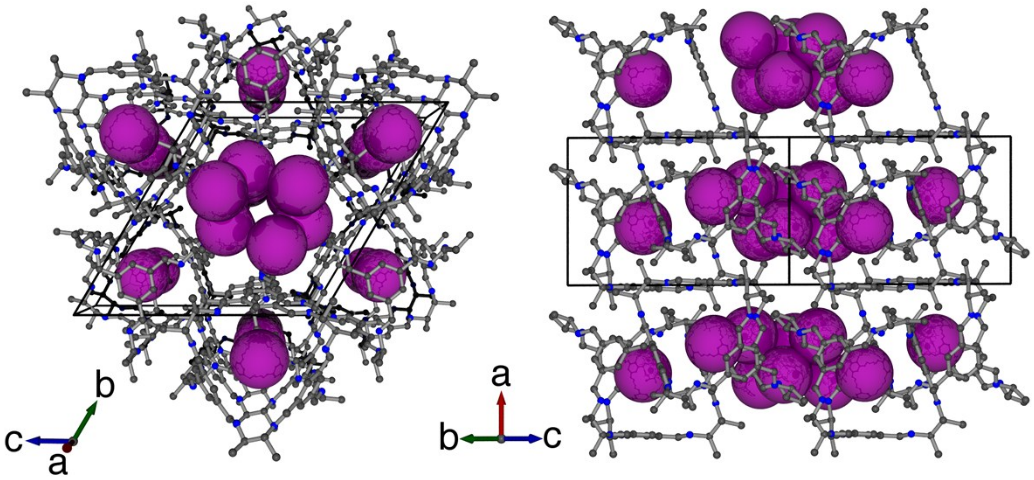

Figure S24. Refined crystal structure of Xe-loaded CC2, **CC2**·2.19Xe (*a* = 10.9666(1), *b* = 18.9072(6), *c* = 19.0061(8) Å, *α* = 120.549(2), *β* = 89.768(4), *γ* = 90.127(3)°, *V* = 3393.8(2) $Å^3$, *P*–1), drawn parallel to (*left*) the [100] direction and (*right*) the [011] direction. Cage molecules are drawn as ball-and-stick and xenon guest molecules in spacefilling representation. Hydrogen atoms are omitted for clarity.

9.0. References

(1) Barbour, L. J. *Chem. Commun.* **2006**, No. 11, 1163.
(2) Holst, J. R.; Trewin, A.; Cooper, A. I. *Nature Chemistry* **2010**, *2* (11), 915.
(3) Tozawa, T.; Jones, J. T. A.; Swamy, S. I.; Jiang, S.; Adams, D. J.; Shakespeare, S.; Clowes, R.; Bradshaw, D.; Hasell, T.; Chong, S. Y.; Tang, C.; Thompson, S.; Parker, J.; Trewin, A.; Bacsa, J.; Slawin, A. M. Z.; Steiner, A.; Cooper, A. I. *Nature Materials* **2009**, *8* (12), 973.
(4) Mastalerz, M. *Chem. Eur. J.* **2012**, *18* (33), 10082.
(5) Hasell, T.; Wu, X.; Jones, J. T. A.; Bacsa, J.; Steiner, A.; Mitra, T.; Trewin, A.; Adams, D. J.; Cooper, A. I. *Nature Chemistry* **2010**, *2* (9), 750.
(6) Smith, W.; Forester, T. R. *J Mol Graph* **1996**, *14* (3), 136.
(7) Forester, T. R.; Smith, W. *Faraday Trans.* **1997**, *93* (17), 3249.
(8) Holden, D.; Jelfs, K. E.; Cooper, A. I.; Trewin, A.; Willock, D. J. *J. Phys. Chem. C* **2012**, *116* (31), 16639.
(9) Melchionna, S.; Ciccotti, G.; Holian, B. L. *Molecular Physics* **1993**, *78* (3), 533.
(10) Willems, T. F.; Rycroft, C. H.; Kazi, M.; Meza, J. C.; Haranczyk, M. *Microporous and Mesoporous Materials* **2012**, *149* (1), 134.
(11) Childs, H. **2013**.
(12) Frenkel, D.; Smit, B. *Understanding molecular simulation: from algorithms to applications*; Elsevier (formerly published by Academic Press), 2002; Vol. 1, pp 1–638.
(13) Dubbeldam, D.; Calero, S.; Ellis, D. E.; Snurr, R. Q. *Molecular Simulation* **2016**, *42* (2), 81.
(14) Sesé, L. M. *Molecular Physics* **2008**, *85* (5), 931.
(15) Jorgensen, W. L.; Maxwell, D. S.; TiradoRives, J. *J. Am. Chem. Soc.* **1996**, *118* (45), 11225.
(16) Levesque, D.; Gicquel, A.; Darkrim, F. L.; Kayiran, S. B. *J. Phys.: Condens. Matter* **2002**, *14* (40), 9285.
(17) Potoff, J. J.; Siepmann, J. I. *AIChE J.* **2001**, *47* (7), 1676.
(18) PELLENQ, R.; Nicholson, D. *Journal of Physical Chemistry* **1994**, *98* (50), 13339.
(19) Parker, J. E.; Potter, J.; Thompson, S. P.; Lennie, A. R.; Tang, C. C. *Materials Science Forum* **2012**, *706-709*, 1707.
(20) Thompson, S. P.; Parker, J. E.; Marchal, J.; Potter, J.; Birt, A.; Yuan, F.; Fearn, R. D.; Lennie, A. R.; Street, S. R.; Tang, C. C. *J Synchrotron Radiat* **2011**, *18* (Pt 4), 637.
(21) Coelho, A. A. *httpwww.topas-academic.net*. 2007.
(22) Petříček, V.; Dušek, M.; Palatinus, L. *Zeitschrift für Kristallographie - Crystalline Materials* **2014**, *229* (5).

(23) Momma, K.; Izumi, F.; IUCr. *J Appl Crystallogr* **2011**, *44* (6), 1272.
